# Collision tumors revealed by prospectively assessing subtype-defining molecular alterations in 904 individual prostate cancer foci

**DOI:** 10.1172/jci.insight.155309

**Published:** 2022-02-22

**Authors:** Jacqueline Fontugne, Peter Y. Cai, Hussein Alnajar, Bhavneet Bhinder, Kyung Park, Huihui Ye, Shaham Beg, Verena Sailer, Javed Siddiqui, Mirjam Blattner-Johnson, Jaclyn A. Croyle, Zohal Noorzad, Carla Calagua, Theresa Y. MacDonald, Ulrika Axcrona, Mari Bogaard, Karol Axcrona, Douglas S. Scherr, Martin G. Sanda, Bjarne Johannessen, Arul M. Chinnaiyan, Olivier Elemento, Rolf I. Skotheim, Mark A. Rubin, Christopher E. Barbieri, Juan Miguel Mosquera

**Affiliations:** 1 Department of Pathology and Laboratory Medicine, Weill Cornell Medicine, New York, New York, USA.; 2 Caryl and Israel Englander Institute for Precision Medicine, Weill Cornell Medicine and NewYork-Presbyterian, New York, New York, USA.; 3 Department of Pathology, Institut Curie, Saint-Cloud, France.; 4 Department of Urology,; 5 Department of Physiology and Biophysics, and; 6 Institute for Computational Biomedicine, Weill Cornell Medicine, New York, New York, USA.; 7 Department of Pathology, Beth Israel Deaconess Medical Center, Boston, Massachusetts, USA.; 8 Harvard Medical School, Boston, Massachusetts, USA.; 9 Michigan Center for Translational Pathology and Department of Pathology, University of Michigan Medical School, Ann Arbor, Michigan, USA.; 10 Department of Pathology and; 11 Department of Molecular Oncology, Institute for Cancer Research, Oslo University Hospital-Radiumhospitalet, Oslo, Norway.; 12 Department of Urology, Akershus University Hospital, Lørenskog, Norway.; 13 Department of Urology, Beth Israel Deaconess Medical Center, Boston, Massachusetts, USA.; 14 Department of Informatics, University of Oslo, Oslo, Norway.

**Keywords:** Genetics, Oncology, Molecular biology, Molecular pathology, Prostate cancer

## Abstract

**BACKGROUND:**

Prostate cancer is multifocal with distinct molecular subtypes. The utility of genomic subtyping has been challenged due to inter- and intrafocal heterogeneity. We sought to characterize the subtype-defining molecular alterations of primary prostate cancer across all tumor foci within radical prostatectomy (RP) specimens and determine the prevalence of collision tumors.

**METHODS:**

From the Early Detection Research Network cohort, we identified 333 prospectively collected RPs from 2010 to 2014 and assessed ETS-related gene (ERG), serine peptidase inhibitor Kazal type 1 (SPINK1), phosphatase and tensin homolog (*PTEN*), and speckle type BTB/POZ protein (*SPOP*) molecular status. We utilized dual ERG*/*SPINK1 immunohistochemistry and fluorescence in situ hybridization to confirm *ERG* rearrangements and characterize *PTEN* deletion, as well as high-resolution melting curve analysis and Sanger sequencing to determine *SPOP* mutation status.

**RESULTS:**

Based on index focus alone, ERG, SPINK1, *PTEN*, and *SPOP* alterations were identified in 47.5%, 10.8%, 14.3%, and 5.1% of RP specimens, respectively. In 233 multifocal RPs with ERG/SPINK1 status in all foci, 139 (59.7%) had discordant molecular alterations between foci. Collision tumors, as defined by discrepant ERG/SPINK1 status within a single focus, were identified in 29 (9.4%) RP specimens.

**CONCLUSION:**

Interfocal molecular heterogeneity was identified in about 60% of multifocal RP specimens, and collision tumors were present in about 10%. We present this phenomenon as a model for the intrafocal heterogeneity observed in previous studies and propose that future genomic studies screen for collision tumors to better characterize molecular heterogeneity.

**FUNDING:**

Early Detection Research Network US National Cancer Institute (NCI) 5U01 CA111275-09, Center for Translational Pathology at Weill Cornell Medicine (WCM) Department of Pathology and Laboratory Medicine, US NCI (WCM SPORE in Prostate Cancer, P50CA211024-01), R37CA215040, Damon Runyon Cancer Research Foundation, US MetLife Foundation Family Clinical Investigator Award, Norwegian Cancer Society (grant 208197), and South-Eastern Norway Regional Health Authority (grant 2019016 and 2020063).

## Introduction

Current consensus clinical guidelines on prostate cancer treatment rely on using the highest grade index lesion for risk stratification (1). Comprehensive molecular analyses of large cohorts of primary prostate adenocarcinoma based on the index lesion, such as The Cancer Genome Atlas (TCGA) and International Cancer Genome Consortium, have identified recurrent molecular alterations and defined distinct molecular subtypes (2, 3).

The promise of genomic data in the clinical setting is to improve risk stratification and guide clinicians in selecting treatment options. Unfortunately, molecular subtype classifiers in primary prostate cancer used in various models have not improved our ability to predict clinical outcomes (4, 5). Primary prostate adenocarcinoma is a multifocal disease (6, 7), and several studies have revealed molecular heterogeneity between spatially distinct foci (8, 9). Therefore, one possibility is that interfocal molecular heterogeneity complicates the use of molecular subtypes at the patient level. Data in advanced prostate cancer cohorts support this possibility because, despite evidence for the monoclonal origin of metastatic lesions (10), one-fourth of metastases may not be clonally linked to the index primary lesion (11).

Prostate cancers can be broadly classified into those with rearrangements in ETS family transcription factors (i.e., *ERG*, *ETV1*, *ETV4*, and *FLI1*) and those without. Up to 60% of primary prostate cancer can be defined by a gene fusion between the androgen-dependent transcription factor transmembrane serine protease 2 (i.e., *TMPRSS2*) and an ETS family oncogene, most frequently ETS-related gene (*ERG*) (12, 13), which can be identified by the surrogate detection of ERG protein overexpression by immunohistochemistry (IHC) (14). Speckle type BTB/POZ protein (*SPOP*) mutation is the most frequent point mutation in primary prostate cancer (~10%) and has been reported as an early clonal event as well as a distinct ETS-negative molecular subclass (6, 15, 16). In addition, the overexpression of serine peptidase inhibitor, Kazal type 1 (SPINK1), protein, although not defining a molecular subtype, has been reported in approximately 10% of primary prostate cancer and is both mutually exclusive from *ETS* rearrangements and associated with *SPOP* mutations (17). Importantly, SPINK1 and ERG protein overexpression can be detected in combination by a dual-color IHC staining, representing a rapid means to visually detect intrafocal and interfocal molecular heterogeneity (18, 19). We previously confirmed the high correlation between ERG IHC and fluorescence in situ hybridization (FISH) (14), supporting that IHC is appropriate to identify tumors of the ERG fusion molecular subgroup and that FISH can be restricted to studies requiring distinction between deletion and insertion rearrangements. Finally, phosphatase and tensin homolog (*PTEN*) deletions have also been identified in up to 20% of primary prostate cancer and have been associated with higher Gleason grade, tumor progression, and early prostate serum antigen (PSA) recurrence (20–22).

Because large-scale studies utilize biorepositories of individual lesions from individual patients, we sought to characterize the molecular subtypes of all foci in the same radical prostatectomy (RP) to better define interfocal heterogeneity. We utilized dual ERG/SPINK1 IHC and FISH to confirm *ERG* rearrangements and assess *PTEN* deletion status, as well as high-resolution melting curve analysis and Sanger sequencing to determine *SPOP* mutation status. These assays allowed us to comprehensively identify molecular alterations in a large multi-institutional cohort of prospectively collected RP specimens.

## Results

### Patient characteristics.

Our initial cohort included 333 patients with median age of 61 and mean PSA of 4.34 ng/mL. The majority of patients had index lesion Gleason grade group 2 (50.3%), pathological stage pT2 (70.0%), lymph node status pN0 (59.5%) disease, with negative surgical margins (76.3%) and no evidence of extraprostatic extension (70.6%) (Table 1).

**Figure 1:**
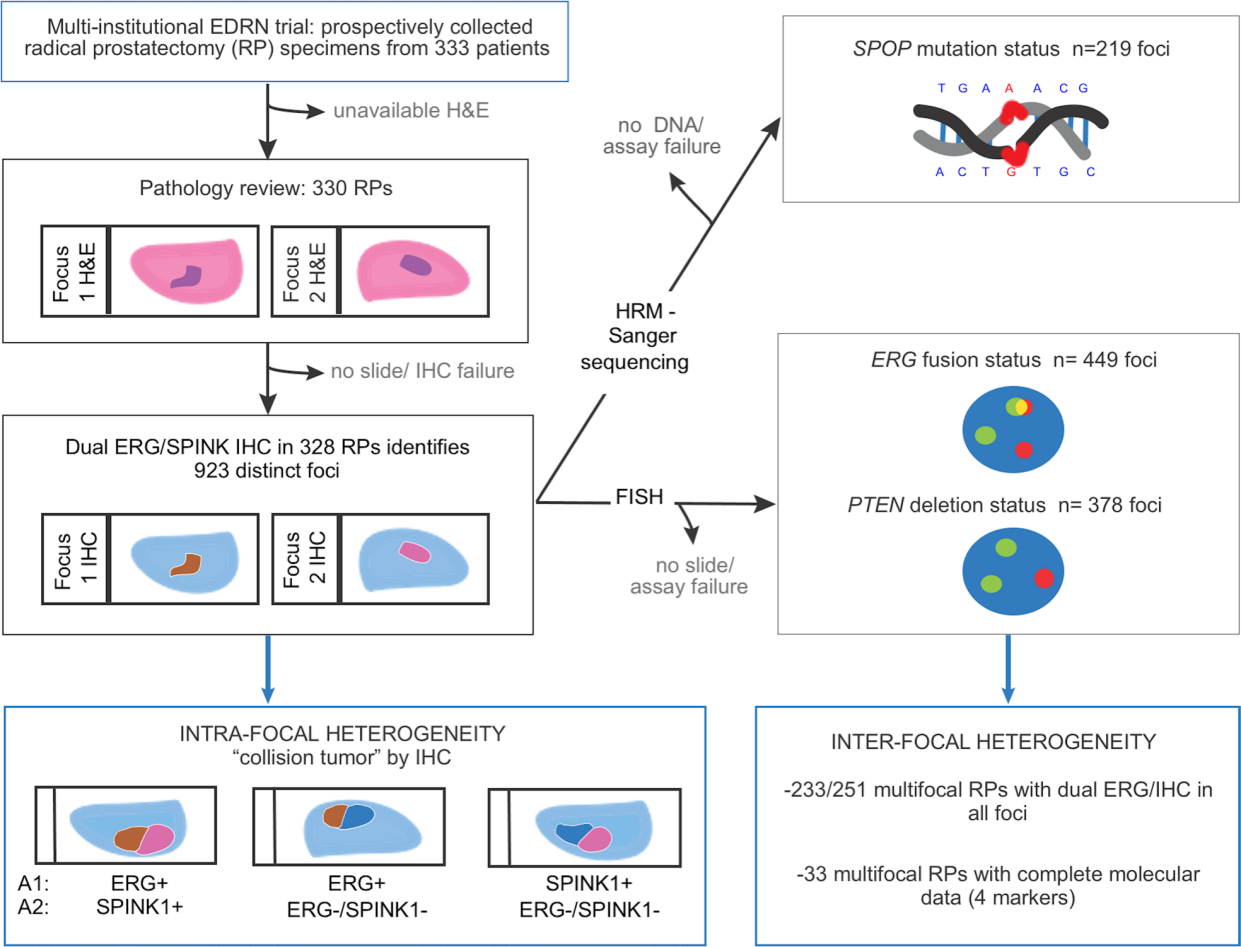
Study workflow of the molecular characterization of RP specimens from the Early Detection Research Network cohort. HRM, high-resolution melting.

**Figure 2:**
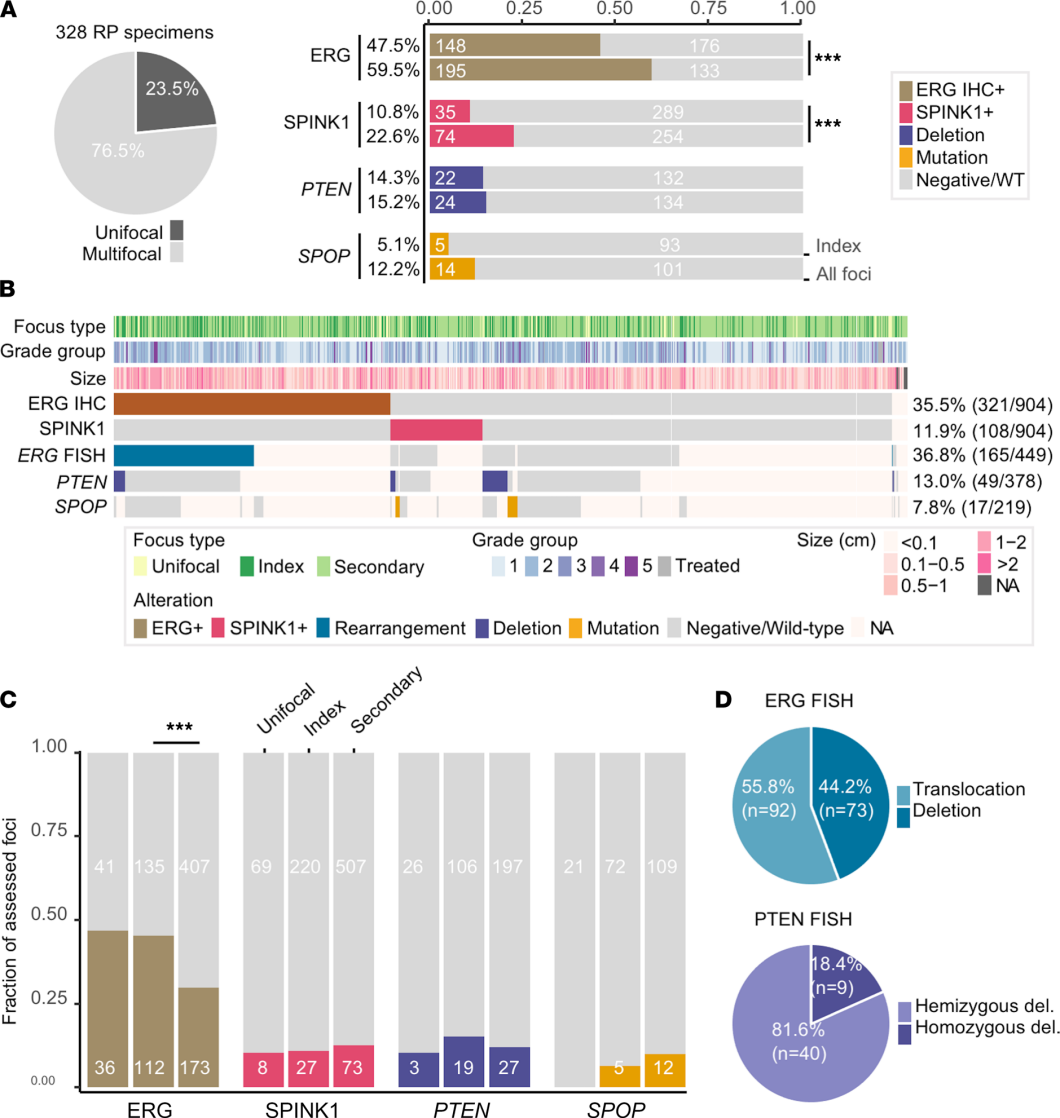
Prevalence of molecular alterations. (**A**) Pie chart displays the proportions of patients with multifocal tumors. Right panel shows bar plots comparing the prevalence of each molecular alteration in the index focus alone or in any focus within each RP specimen. (**B**) Molecular alteration prevalence across all tumor foci (*n* = 923) and pathological characteristics. (**C**) Bar plots comparing the prevalence of each molecular alteration when considering the type of focus (unifocal, index, or secondary focus). (**D**) Percentages of type of ERG rearrangement and PTEN deletion. ***P < 0.001, χ^2^ test.

Five RP specimens were excluded from further analysis because of unavailable H&E slides for pathological review or unavailable unstained slides for molecular alteration assessment (Figure 1). Based on combined H&E and IHC review, 923 tumor foci were identified in the 328 available RPs. Seventy-seven patients (23.5%) had a single tumor focus, and 251 (76.5%) had multifocal tumors (98 or 29.9% had 2 focim, and 153 or 46.6% had at least 3 foci) (Figure 2A). No correlation of number of tumor foci per RP specimen was seen with any clinical or pathological parameters (data not shown). While tumor foci measured up to 3.5 cm on a glass slide, the median focus size was 0.6 cm. Of the 913 foci with an available Gleason score, the vast majority were classified as grade group 1 (*n* = 384 or 42.1%) or 2 (*n* = 393 or 43.0%) (Figure 2B).

### Molecular alteration frequencies.

To evaluate molecular alteration frequencies on a per patient basis, we classified each prostatectomy specimen by the presence of an alteration (a) solely in the index lesion or (b) in at least 1 of all identified tumor foci within an RP specimen. Based on the index lesion, ERG, SPINK1, *PTEN*, and *SPOP* molecular alterations occurred in 47.5%, 10.8%, 14.3%, and 5.1% of patients, respectively (Figure 2A). When considering all tumor foci, the frequency of ERG (59.5%, *P* = 5.8 × 10^–5^) and SPINK1 (22.6%, *P* = 8.9 × 10^–5^) overexpression was significantly higher than when considering the index focus alone. *PTEN* and *SPOP* mutations occurred in 15.2% and 12.2% of prostatectomies when considering at least 1 altered focus within an RP specimen.

Out of 923 foci, IHC results were unavailable for 19 foci (2.1%) because of tumor exhaustion on subsequent IHC slide or assay failure. ERG and SPINK1 overexpression were detected in 35.5% (*n* = 321) and 11.9% (*n* = 108), respectively, of the 904 foci evaluated by IHC (Figure 2B). As expected, ERG and SPINK1 overexpression were mutually exclusive at each tumor focus. We did identify 1 ERG^+^ focus (0.1%) with simultaneous SPINK1 expression within the same tumor cells in less than 5% of the focus. *ERG* rearrangement, *PTEN* deletion, and *SPOP* mutation status was available in a subset of 449, 378, and 219 foci, respectively.

To further explore the differences in molecular alteration frequencies by tumor focus type, we classified each focus as unifocal, index lesion, or secondary lesion on each prostatectomy specimen. In RP specimens with multifocal tumors, there were significantly more ERG^+^ index tumors compared with secondary tumors (45.3% vs. 29.8%, *P* = 2.5 × 10^–5^) (Figure 2C). No significant difference in alteration frequency between index foci and secondary foci was found for SPINK1, *PTEN*, or *SPOP*.

All 165 ERG^+^ foci of a total of 449 foci evaluated by FISH were confirmed to be *ERG* rearranged, 55.8% through translocation and the remaining 44.2% through deletion (Figure 2D). No ERG^–^ focus by IHC was found to be *ERG* rearranged by FISH. *SPOP* missense mutations occurred in 7.8% of foci (17/219) and were mutually exclusive from ERG overexpression. *SPOP* mutations were most commonly F133L, followed by F133V, F102V, F102G, F102G, W131G, and D130N. *PTEN* deletion was detected in 13.0% (*n* = 49) of 378 tumor foci examined, with 9 homozygous (18.4%) and 40 heterozygous (81.6%) deletions.

### Molecular alterations and clinical characteristics.

Associations between baseline clinical characteristics and molecular alterations were determined based on considering all foci or only the index lesion in each prostatectomy specimen. When considering all foci, there were no significant differences in clinical characteristics when comparing patients with and without *PTEN* deletions as well as those with and without *SPOP* mutations. Patients with ERG overexpression were more likely to be recruited from Weill Cornell Medicine (WCM) (*P* = 0.003), and patients with SPINK1 overexpression were less likely to be Black (*P* = 0.001). These associations remained statistically significant when considering a Bonferroni-corrected α of 0.0125. When considering only the index focus, there were no significant associations between clinical characteristics and subtype-defining molecular alterations.

### Interfocal heterogeneity.

**Figure 3:**
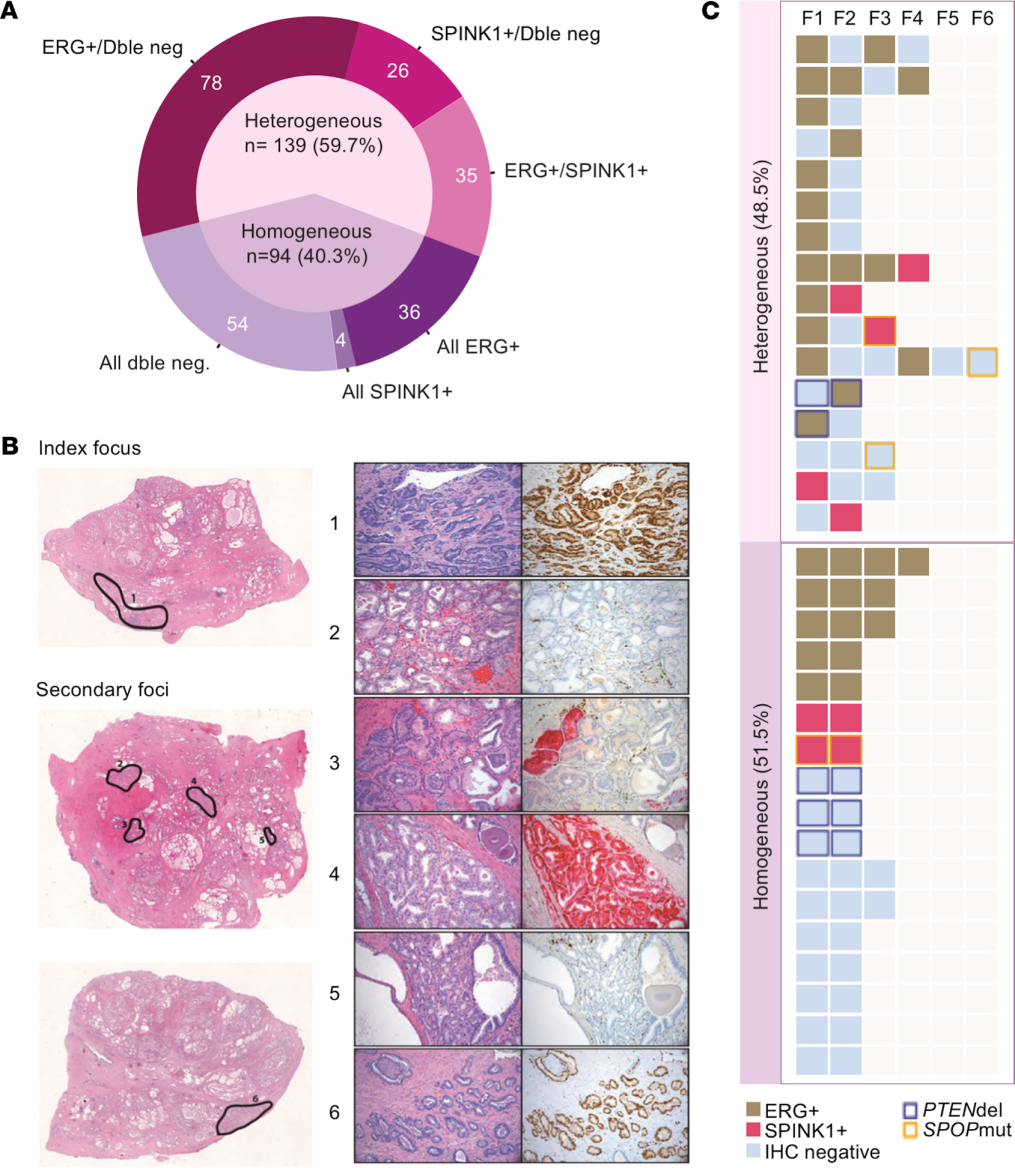
Interfocal heterogeneity of molecular alterations. (**A**) Proportion of multifocal cases with interfocal heterogeneity based on ERG/SPINK1 IHC. (**B**) Representative specimen with heterogeneity across 6 tumor foci that are ERG^+^ (foci 1, 6), SPINK1^+^ (foci 3, 4), or double negative (foci 2, 5). Original magnification, 20× (left), 100× (right). (**C**) Representation of each focus (columns) of the 33 multifocal cases (lines) in which alterations of ERG, SPINK1, PTEN, and SPOP were evaluated in all foci.

Of the 251 prostatectomy specimens harboring multiple tumor foci, ERG/SPINK1 IHC was performed on all foci in 233 (92.8%) specimens to be able to evaluate for interfocal heterogeneity. Heterogeneous molecular alterations were identified in 139 RPs (59.7%) (Figure 3, A and B). Among heterogeneous cases, 78 (56.1%) had a combination of ERG^+^ and double-negative foci, 35 (25.2%) had ERG^+^ and SPINK1^+^ foci, and 26 (18.7%) had SPINK1^+^ and double-negative foci.

Molecular status for all 4 markers, *ERG*, SPINK1, *PTEN*, and *SPOP*, was available in all foci of 33 RP specimens. We found 16 (48.5%) had discordant molecular alterations between foci (Figure 3C). ERG positivity was again the main determinant of heterogeneity, as 81.2% (*n* = 13) of heterogeneous cases had at least 1 ERG^+^ focus.

### Intrafocal heterogeneity.

**Figure 4:**
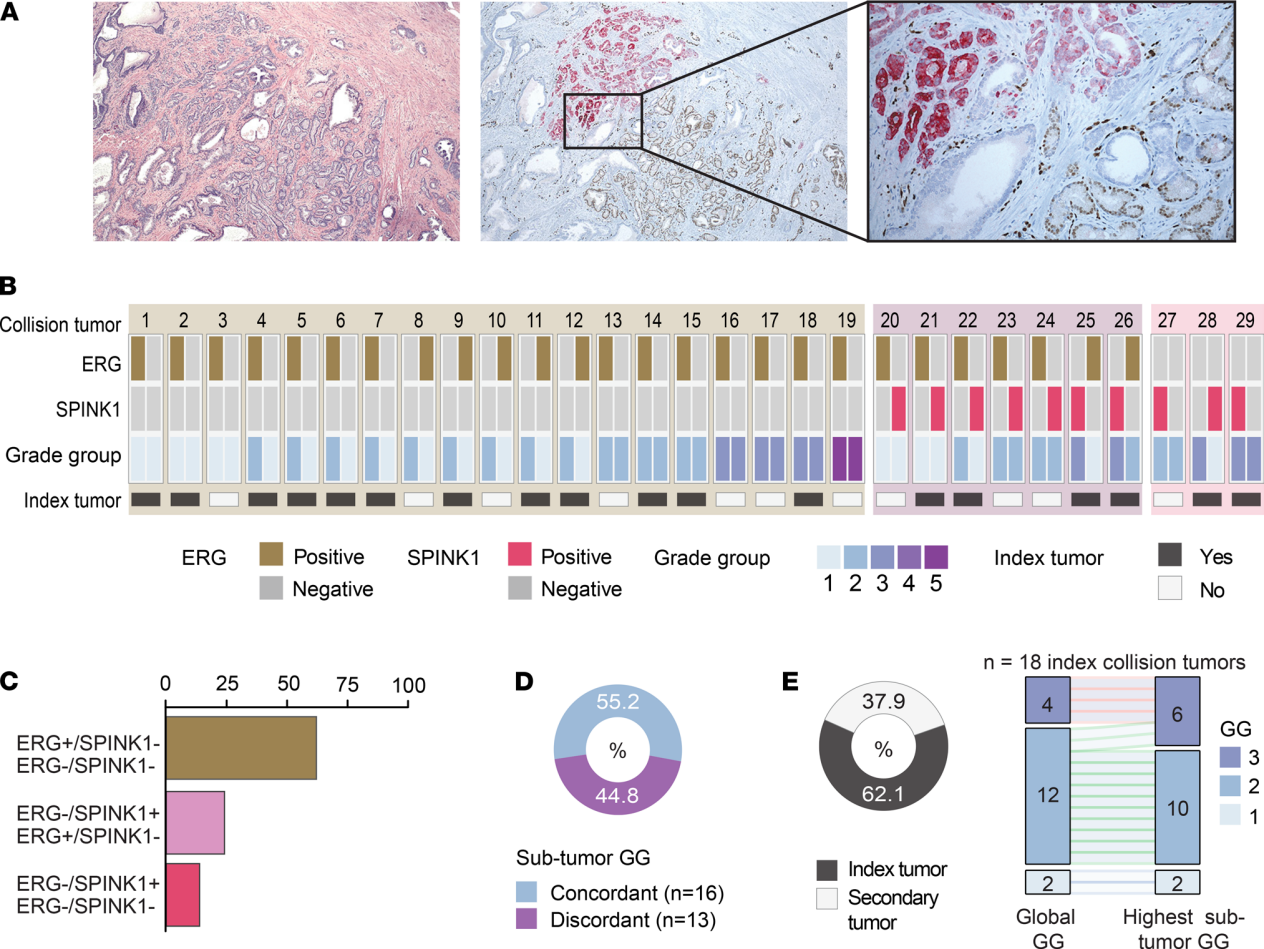
Characterization of intrafocal heterogeneity. (**A**) Representative tumor focus with 2 colliding subtumor areas: ERG overexpression (brown) and SPINK1 overexpression (pink). Original magnification, 40× (left and center), 200× (right). (**B**) Molecular alterations present in each collision tumor and pathological characteristics. (**C**) Frequency of alterations found in collision tumors. (**D**) Frequency of discrepancy in Gleason grade group between subtumor areas. (**E**) Frequency of collision tumors occurring in index lesion and frequency of Gleason group grade (GG) reclassification.

Of the 310 RP specimens (multifocal and unifocal) examined by dual ERG/SPINK1 IHC in all tumor foci, we identified collision tumors in 9.4% (*n* = 29) of patients (Figure 4, A and B). A collision tumor was defined as a single circumscribed tumor focus composed of 2 colliding, clonally distinct subpopulations. The discordance in molecular status was noted to be an ERG^+^ area with an adjacent double-negative area in 65.5% (*n* = 19) of cases, an ERG^+^ area adjacent to a SPINK1^+^ area in 24.1% (*n* = 7) of cases, or a SPINK1^+^ area with an adjacent double-negative area in 10.3% (*n* = 3) of cases (Figure 4C). Nearly half (44.8%, *n* = 13) of the collision tumors had discordant Gleason score/grade group between subtumor areas (Figure 4D). The phenomenon of collision tumor occurred in the index tumor in 62.1% (*n* = 18) of the cases, of which 2 cases (11.1%) demonstrated upgrading when reclassified according to the highest subtumor Gleason score/grade group (Figure 4E). In these 2 cases, the initial lesion identified by H&E was classified as a grade group 2 lesion. However, the subtumor areas identified by IHC revealed the presence of a smaller, higher grade lesion (grade group 3).

### Correlation with clinical prognosis.

**Figure 5:**
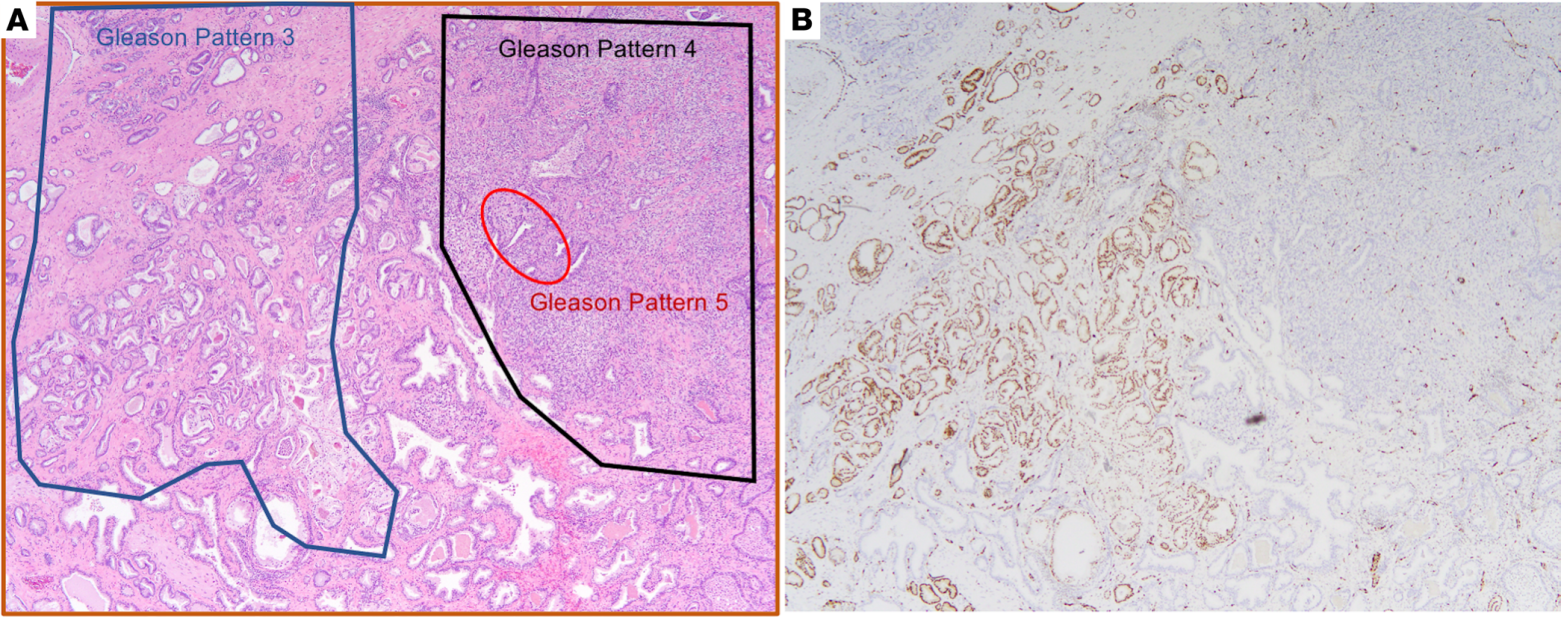
Representative case of pathological discordance within a collision tumor leading to Gleason score upgrade. (**A**) Initial histopathology review with diagnosis of Gleason score 4 + 3 = 7 with tertiary pattern 5. (**B**) Subsequent IHC, which suggests the presence of an ERG-positive grade group 1 focus (left) colliding with an ERG-negative grade group 5 focus (right). Original magnification, 40×.

We did identify some patients who were inaccurately classified into lower Gleason score/grade groups through conventional clinical H&E pathological review. To illustrate this, we present the case of a 61-year-old otherwise healthy man with serum PSA 12.1 ng/mL and magnetic resonance imaging/ultrasound fusion-guided prostate biopsy showing Gleason grade group 3 disease. He underwent robotic-assisted laparoscopic prostatectomy with initial H&E histopathology diagnosis of prostate adenocarcinoma Gleason score 4 + 3 = 7 with tertiary pattern 5 disease (Figure 5A). However, given the disparate cytologic and architectural features, we performed ERG IHC and discovered the presence of a lower grade ERG-positive tumor adjacent to a higher grade ERG-negative tumor (Figure 5B). Given these findings, he was ultimately classified as having a dominant grade group 5 (Gleason score 4 + 5 = 9) lesion with cribriform growth and extraprostatic extension and an adjacent secondary grade group 2 (Gleason score 3 + 4 = 7) lesion.

## Discussion

We performed a comprehensive pathological review of over 300 RPs from a prospectively collected multi-institutional cohort and mapped and graded each tumor focus based on H&E examination and molecular classifiers (*ERG* rearrangement, SPINK1 overexpression, *PTEN* deletion, and *SPOP* mutation). Most patients (76.5%) had multifocal disease, with most foci being grade group 1 (42.1%) or 2 (43.0%) disease. When categorizing based on the index focus, ERG, SPINK1, *PTEN*, and *SPOP* alterations were identified in 47.5%, 10.8%, 14.3%, and 5.1% of RP specimens. However, there was a significantly higher frequency of ERG (59.5%) and SPINK1 (22.6%) overexpression when incorporating molecular subtypes of all foci on an RP specimen basis. Similar to our findings, the TCGA analysis utilizing the dominant focus to classify patients also found that 46% of patients with primary prostate adenocarcinoma undergoing RP can be classified as *ERG* fusion gene subtype (2). Prior data also showed that the percentage of *ERG* subtype decreases when doing a comprehensive analysis of all tumor foci (23). Our analysis shows that only 35.5% (321/904) of all tumor foci displayed ERG IHC staining and that 36.8% (165/449) of all tumor foci were *ERG* FISH positive. This suggests that small, incidentally detected tumors are less likely to have *ERG* rearrangements and highlights the difficulty of characterizing lesions in a comprehensive analysis that would otherwise go undetected and uncharacterized.

Of the 33 RP specimens with molecular status for ERG, SPINK1, *PTEN*, and *SPOP*, 16 (48.5%) had discordant molecular alterations between foci. Although we observed higher frequency of molecular alterations than previously reported (6, 12), we attribute this to our method of evaluating every tumor focus in the RP specimens, not just the dominant one. Beyond interfocal heterogeneity, the utility of molecular classification has also recently been challenged by intrafocal heterogeneity, with a report of approximately 12% of tumor foci having conflicting molecular subtype classifications between different samples of the same focus (23). Our group previously identified the presence of 2 immediately adjacent, but distinct, tumor foci found to have *ERG* rearrangement and F133V *SPOP* mutation (16). We hypothesized that this phenomenon defines the intrafocal molecular heterogeneity previously observed and contributes to the difficulty in using molecular subtypes at the patient level for risk stratification. We identified 29 (9.4%) RP specimens with collision tumors, which were defined as a single circumscribed focus composed of 2 colliding, clonally distinct subpopulations. Overall, only 2 of these cases had Gleason score/grade group discordance resulting in upgrading when considering subtumor areas. Additionally, although 55.2% (*n* = 16) of the collision tumors had concordant Gleason grade group between subtumors, these likely represented clonally distinct, independent tumors, and future research should be done to better characterize the aggressiveness of these subtumors.

Because we utilized dual ERG/SPINK1 IHC, and 37.9% (22/58) of the collision tumors were Gleason grade group 3, we cannot eliminate the possibility that some of these represent clonally similar populations undergoing an initial somatic expansion of an *ERG* rearrangement area. Previous data suggested that *ERG* fusions are detected in areas of high-grade prostatic intraepithelial neoplasia and likely represent an early event that precedes other chromosome-level alterations found in prostate cancer (24, 25). This supports our hypothesis that collision tumors represent clonally distinct regions, though future testing should be done to validate these findings.

To our knowledge, this is the most comprehensive analysis on the molecular characteristics of all tumor foci and the first published report on the phenomenon of collision tumors in primary prostate cancer. The rates we detected are similar to previously reported rates of intrafocal genomic heterogeneity (9.4% vs. 12%) based on sampling different areas of the same tumor focus (24). In almost half the cases (44.8%) of collision tumors, the Gleason score/grade group between the 2 subtumors were discordant. Clinically, this is concerning for potentially misclassifying patients as lower risk when a higher Gleason score/grade group subtumor area is diluted by a neighboring lower grade area. However, we did not detect a difference in biochemical recurrence-free survival in our limited cohort.

Regardless, our current data do suggest that this phenomenon is important to consider for future genomic studies as approximately 10% of patients may have a collision tumor. We propose that future studies utilize a screening test, such as dual ERG/SPINK1 IHC, as an adjunct to H&E staining to characterize the presence of collision tumors, which may significantly alter results of genomic heterogeneity. In addition, this screening tool may also be considered in clinical histopathology practice if there are disparate cytologic and architectural features within what appears to be a single H&E focus, to rule out the possibility of a large area of lower grade tumor masking a smaller area of higher grade disease, and to avoid Gleason score “dilution” (Figure 5).

There are several limitations in this study. First, we were unable to perform a comprehensive, unbiased molecular characterization of each tumor focus due to the sheer volume of cases. As such, we mainly utilized the broad categorizations of ERG overexpression, SPINK1 overexpression, and ERG^–^SPINK1^–^. Because of the difficulty in extracting sufficient quality genomic DNA from very small tumor foci, we were limited in our ability to characterize all tumor foci *SPOP* mutation status. However, the HRM assay we utilized allows for detection of a significant shift in the HRM curve in cases with as low as 5% mutated DNA (15, 16). Taking all of this into account, our data define clonal heterogeneity based on subtype-defining molecular alterations across a large cohort of prostatectomy specimens through systematic pathological analysis; however, it does not reflect the true clonal relationship that would be revealed by whole-genome sequencing. Second, our conclusions can only be generalized to patients undergoing RP as our data did not characterize the patient population undergoing active surveillance or radiation therapy. Finally, our cohort of collision tumor patients with follow-up data (*n* = 14) was limited given the small number of patients with collision tumors. We therefore acknowledge that, at the moment, this phenomenon may be more relevant for research workflow rather than clinical application.

In conclusion, interfocal molecular heterogeneity is found in approximately 60% of RP specimens. The phenomenon of collision tumors was identified in approximately 10% of patients with primary prostate cancer. Sampling within the distinct clonal populations of a collision tumor may explain the previously reported intrafocal genomic heterogeneity. Further studies should be conducted to determine whether these levels of heterogeneity may affect the use of molecular classifications in models predictive of clinical and pathological outcomes.

## Methods

### Study population.

Patients were from 3 participating institutions, NewYork-Presbyterian–WCM, University of Michigan Medical School, and Beth Israel Deaconess Medical Center. Available clinical characteristics included patient age at diagnosis, race, preoperative PSA, and date of surgery. Race was based on participant self-classifications and was designated as unknown if none was selected.

### General pathology review.

The study workflow to characterize intrafocal and interfocal molecular heterogeneity in each RP is summarized in Figure 1. Prostatectomy H&E-stained slides were reviewed by 4 genitourinary pathologists. In each case, every spatially distinct tumor focus was annotated for Gleason score, grade group, and tumor size. As in the previous review from the International Society of Urological Pathology consensus conference (26), a formal definition of the identifying features of the dominant/index tumor remains undecided. In this study, the focus with the highest Gleason score/grade group (or stage when not organ confined) defined the index tumor focus. In cases with multiple organ-confined foci of the same Gleason score, the largest was considered the index focus. Other available pathology features included prostate weight, pathological stage (pT), lymph node status (pN), lymphovascular invasion, extraprostatic extension, perineural invasion, seminal vesicle invasion, and surgical margin status. A representative formalin-fixed, paraffin-embedded (FFPE) block was selected for each tumor focus for further molecular characterization. Five RP specimens were excluded from further analysis due to unavailable H&E slides for pathological review or unavailable unstained slides for molecular alteration assessment (final *n* = 328).

### ERG and SPINK1 status.

Dual ERG/SPINK1 IHC was performed on 4-μm-thick unstained slides from the representative block of each tumor focus using a monoclonal rabbit anti-ERG primary antibody from Ventana Medical Systems (clone EPR3864) and a mouse primary anti-SPINK1 4D4 antibody (Abnova). IHC was performed on the BenchMark ULTRA automated staining system (Ventana Medical Systems), as previously described (14). Tumor foci were considered ERG^+^ if they displayed diffuse moderate (2+) or intense (3+) nuclear staining in the presence of a positive control (3+ stained endothelial cells). A tumor focus with moderate (2+) or intense (3+) cytoplasmic staining in at least 5% of tumor cells was considered SPINK1^+^. Both ERG^+^ and SPINK1^+^ controls (*ERG*-rearranged prostate cancer and pancreatic tissue, respectively) and negative controls (benign prostate tissue) were included in each run of the Ventana autostainer.

Additionally, *ERG* rearrangement status was evaluated in a subset of cases by performing a dual-color break-apart interphase FISH on a separate 4-μm-thick unstained slide of each tumor focus, as previously described (13). We used red-labeled (BAC clone RP11-24A11) and green-labeled probes (BAC clone RP11-372O17), which span the centromeric and telomeric regions of *ERG*, respectively.

### Identification of collision tumors.

A collision tumor was defined as a single circumscribed tumor focus composed of 2 colliding, clonally distinct subpopulations, i.e., with discrepant ERG and/or SPINK1 staining. Collision tumors were categorized as ERG^+^/double negative, SPINK1^+^/double negative, or ERG^+^SPINK1^+^ according to intrafocal heterogeneity type (Figure 1). Each molecularly distinct area was then further considered as an individual focus for our analyses regarding molecular alteration frequency and distribution, and each area was annotated for individual size and Gleason score/grade group.

### SPOP status.

*SPOP* mutation status was determined based on methods previously described by our group (15). Briefly, after macrodissection of unstained slides for each focus and deparaffinization, DNA was extracted using Promega Maxwell 16 FFPE Tissue LEV DNA Purification Kit (AS1130) on the Promega Maxwell 16 MDx Instrument. Following pre-PCR target enrichment of exons 6 and 7, a mutational screening assay using HRM analysis was performed. For a subset of HRM-positive cases with sufficient high-quality DNA (*n* = 11), PCR products were purified and analyzed by Sanger sequencing to confirm the mutational status and to locate the mutated residue. PCR assay setup, cycling conditions, HRM assay, and analysis are described in Blattner et al. (15). PCR was performed using a LightCycler 480 II (Roche Diagnostics).

### PTEN status.

*PTEN* deletion status of a subset of tumor foci was detected using FISH performed on representative unstained slides that were 4 μm thick. A *PTEN*-specific red-labeled probe (BAC clone CTD-2047N14) and a reference green-labeled probe located at 10q25.2 (23.5 megabase pairs distal to *PTEN*; BAC clone RP11-431P18) were used. Tumor foci were considered *PTEN* deleted if 1 (hemizygous deletion) or 2 (homozygous deletion) copies of the gene-specific probe were absent, in the presence of 2 reference signals per nucleus. For all FISH evaluations, at least 100 cancer nuclei were evaluated per tumor focus using a fluorescence microscope (Olympus BX51, Olympus Optical).

### Statistics.

All statistical analyses were performed with SPSS 23 (SPSS Inc., IBM Corp.). Fisher’s exact test and χ^2^ test were used to evaluate the association between categorical variables. All tests are 2 sided and a *P* *<* 0.05 was considered statistically significant.

### Study approval.

RP specimens from 333 patients with clinically localized prostate cancer were prospectively collected between 2010 and 2014 after obtaining written consent and enrolling patients in the Early Detection Research Network study (NCI 5U01 CA111275). The study was approved by WCM Institutional Review Board (11157-05).

## Author contributions

CEB and JMM designed the research studies. JF, PYC, HA, BB, KP, HY, SB, VS, JS, MBJ, JAC, ZN, CC, TYM, UA, MB, and KA conducted experiments, acquired data, and analyzed the data. JF and PYC wrote the manuscript. DSS, MGS, BJ, AMC, OE, RIS, MAR, CEB, and JMM helped revise the manuscript. Authorship among first authors was assigned based on number of years involved in the project.

## Supplementary Material

ICMJE disclosure forms

## Figures and Tables

**Table 1:**
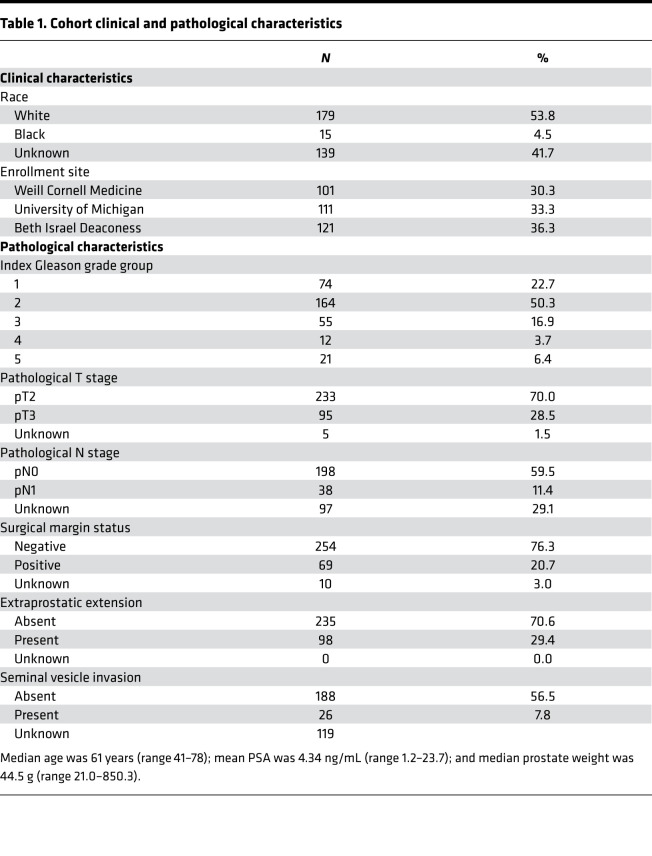
Cohort clinical and pathological characteristics

## Version 1 01/20/2022

In-Press Preview

## Version 2 02/22/2022

Electronic publication
